# Effect of BMI on blood value of patients on HCG day with IUI treatment

**DOI:** 10.1186/s12905-020-00963-1

**Published:** 2020-05-14

**Authors:** Li-Ting Wang, Cheng-Xiang Wang, Hong-Liang Sun, Xue Wang, Xue-Feng Li, Yan-Lin Wang, Qing-Chun Li

**Affiliations:** Department of Reproductive Medicine, Binzhou Medical University Hospital, Binzhou, China

**Keywords:** Intrauterine insemination, Body mass index, E_2_, P, LH

## Abstract

**Background:**

This study aims to analyze the effect of the body mass index (BMI) on E_2_, P and LH values in females who received intrauterine insemination (IUI) treatment on human chorionic gonadotropin (HCG) day.

**Methods:**

A total of 2319 cycles of IUI-assisted pregnancy treatment were selected in our hospital. Based on the BMI, female infertility patients are divided into three groups: normal weight group, overweight and obese group.

**Results:**

For patients with natural cycles and ≤ 35 years old, there were 440, 178 and 197 cases in the three groups, respectively. For patients with natural cycles and > 35 years old, there were 90, 83 and 81 cycles in the three groups, respectively. For patients with induced ovulation cycle and ≤ 35 years old, there were 425, 203 and 516 cases in the three groups, respectively. For patients with induced ovulation cycle and > 35 years old, there were 26, 26 and 54 cases in the three groups, respectively.

**Conclusion:**

When a patient is ≤35 years old, the BMI affects the E_2_, LH and *P* values on the day of artificial insemination. However, the BMI is negatively correlated with E_2_, LH and P in IUI on HCG day. After controlling for age and assisted pregnancy, the correlation analysis revealed that the BMI is negatively correlated with hormone E_2_ and LH. The higher the BMI was, the lower the levels of hormones E_2_, LH and P became. However, in the present study, the BMI did not significantly improve the clinical pregnancy rate of patients who received IUI.

## Background

With the rapid development of the economy, the pace of people’s life speeds up, material living standard improves, and obesity becomes a global public health problem. It has been proven by growing evidence that obesity can result in infertility, such as menstrual cycle disorder, reduced fertility, abnormal ovulation, high androgen levels and insulin resistance [1–5]. The maintenance of normal reproductive function in females is affected by various factors, and the influence of BMI on assisted reproduction has become a hot topic for debate [6, 7]. Studies conducted abroad have reported that obese women have low fertility for both the natural state and assisted-reproductive technology treatment [8, 9].

There are many reasons for the lower fertility of obese women. To date, the most widely accepted mechanism is that the balance of the hypothalamic-pituitary-ovarian (HPO) axis is disrupted in obese women. Insulin resistance and leptin resistance often occurs [10–14], and the level of free fatty acids in the blood of obese people is higher, which can directly inhibit Gn synthesis. Furthermore, the leptin secreted by fat cells interferes with the HPO axis, thereby affecting the Gn release, and resulting in decreased levels of estradiol (E_2_), luteotropic hormone (LH) and progesterone (P) in serum [15]. The study conducted by Souter et al. [16] revealed that the decrease in serum estradiol and LH levels in obese people may be correlated to the increase in concentration of anti-gonadal hormone, which is similar to the leptin in serum and follicular fluid. In addition, leptin can inhibit the E_2_ secretion by human cells, and regulate the generation of steroid hormone.

The disorder of the HPO axis affects the regularity of the menstrual cycle, and inhibits the occurrence of ovulation [17]. Obesity not only affects the menstruation and ovulation of women during their reproductive period, but also to some extent damages the egg quality and uterine receptivity [18]. It was found in a study on obesity and in vitro fertilization outcomes that the number of mature eggs of women with a BMI of more than 30 kg/m^2^ significantly decreased, suggesting that obesity is an independent risk factor that hinders egg maturation [19]. In the normal menstrual cycle, progesterone P is generally not secreted at the follicular stage. Before ovulation, the granulosa cells of a mature follicle are luteinized under the action of LH ovulation peak, and begins to secrete a very small amount of progesterone [20]. After ovulation, P is mainly generated by granular luteal cells and follicular membrane luteal cells. These are transported by blood to the target cells, and combines with the corresponding receptor to play certain roles [20]. The main roles are, as follows: (1) make the endometrium in the proliferating stage transform into a secretory membrane, and prepare for the fertilized egg implantation and embryonic development in the later stage; (2) reduce the excitability of uterine smooth muscles and its sensitivity to oxytocin, thereby inhibiting uterine contraction, which is conducive to embryonic development, and intrauterine fetal growth and development. A retrospective analysis of a large sample in China revealed that excessively high P on the first day after HCG day affects the oocyte retrieval rate, but does not affect the embryo quality, embryo implantation rate and clinical pregnancy rate [21].

In the present study, a total of 2319 cycles of intrauterine insemination (IUI) and assisted pregnancy in the Reproductive Center of Binzhou Medical University Affiliated Hospital were selected as the study object, in order to investigate the effect of body mass index (BMI) on E_2_, P and LH on HCG day and pregnancy outcome for women of different ages.

## Methods

### General information

A total of 2319 cycles of IUI conducted in the Reproductive Center of Binzhou Medical University Affiliated Hospital from 2014 to 2016 were retrospectively analyzed. Inclusion criteria: (1) patients within 20–45 years old, (2) patients with a BMI of 18.5–35.1 kg/m^2^; (3) patients without uterine malformation, and (4) patients with salpingography or hysteroscopy results showing that the fallopian tube is unobstructed at one side at least. Exclusion criteria: infertile women with polycystic ovary syndrome, endometriosis, uterine leiomyoma, uterine malformation, and a history of ovarian surgery. Due to the different BMI characteristics for different populations, the present study adopted the BMI grading standard, which was formulated by the World Health Organization (WHO) for the Asia Pacific region. This was suitable for the physical characteristics of Chinese subjects. In addition, the influence of the BMI grading standard on blood value on HCG day and pregnancy outcome during the IUI treatment was also discussed.

In order to exclude the effects of different endometrial preparation regimens and age on the results of the present study, the 2319 cycles of subjects are divided into the following groups: (1) IUI with natural cycle and < 35 years old; (2) IUI with natural cycle and > 35 years old; (3) IUI with ovulation induction cycle and < 35 years old; (4) IUI with ovulation induction cycle and > 35 years old. According to the WHO reference standard for Asian women’s BMI, the above four groups were further divided into three groups: normal weight group (18.5 kg/m^2^ ≤ BMI ≤ 23.0 kg/m^2^), overweight group (23 kg/m^2^ ≤ BMI < 25 kg/m^2^), and obese group (25 kg/m^2^ ≤ BMI ≤ 35.1 kg/m^2^). These patients were divided into three groups according to the order of BMI grouping.

### Methods

Follicle monitoring: the natural cycle was adopted for patients with regular menstruation, while the CC/LE/CC + HMG/LE + HMG/HMG/FSH + HMG ovulation induction cycle was adopted for patients with ovulation disorders. When the follicle diameter was more than 14 mm, vaginal ultrasound monitoring was performed daily, and the ovulation was evaluated. When the dominant follicle diameter was more than 16 mm, the LH peak value was detected using the LH semi-quantitative ovulation test paper every day to predict the ovulation. Then, according to the predicted results, the dosage and time of HCG for ovulation induction were determined. The levels of E_2_, P and LH in serum were monitored on HCG day. These patients were treated with IUI on the day after HCG injection, an all patients were treated with progesterone after IUI.

Semen preparation: The abstinence for the husband was approximately 3 or 7 days, the sperm was extracted by masturbation and placed in a sperm cup, and the semen is treated by density gradient centrifugation. After optimization, the sperm suspension was 0.5 ml, and the number of forward moving sperms was more than 10,000. IUI was subsequently performed.

Insemination methods: These patients were placed in the lithotomy position, a saline cotton ball was used to wash the vagina and cervix, the processed semen was extracted using a one-off artificial insemination tube, and this was slowly injected into the patient’s uterine cavity. The head low and buttocks high posture was maintained for 30 min, and the patient was discharged from the hospital. Luteal support was performed for all patients with IUI after the procedure.

Clinical pregnancy determination: Urine HCG or blood HCG were examined at 14 days after the IUI, in order to determine whether the patient was pregnant. The intrauterine pregnancy sac was observed by vaginal ultrasonography between the fourth week and fifth week after IUI, and all subjects were followed up after the IUI.

### Statistics analysis

The data were organized using Excel 2007, and the SPSS 22.0 software was used for the statistical analysis. Normally distributed data were presented as^−^x ± standard deviation (SD), while non-normally distributed data were presented in M (Q25-Q75). The normally distributed data with homogeneous variance were compared among multiple groups using one-way analysis of variance, while non-normally distributed measurement data or data with heterogeneous variance were compared using the Kruskal-Wallis non-parametric H-test. Counting data were presented in frequency, and chi-square test was used for comparison. Simultaneously, Pearson’s correlation was used for the correlation analysis of normally distributed data, while Spearman’s correlation was used for the correlation analysis of non-normally distributed data. If the *P*-value was < 0.05, the difference was statistically significant.

## Results

### General information

The 2319 cycles of subjects are divided into the following groups: (1) 815 cases underwent IUI with a natural cycle, < 35 years old; (2) 254 cases underwent IUI with a natural cycle, > 35 years old; (3) 1144 cases underwent IUI with an ovulation induction cycle, < 35 years old; (4) 106 cases underwent IUI with an ovulation induction cycle, > 35 years old. According to the WHO reference standard for Asian women’s BMI, the above four groups were further divided into three subgroups: normal weight group (18.5 kg/m^2^ ≤ BMI ≤ 23.0 kg/m^2^), overweight group (23 kg/m^2^ ≤ BMI < 25 kg/m^2^), and obese group (25 kg/m^2^ ≤ BMI ≤ 35.1 kg/m^2^).

### Comparison of hormone and pregnancy for patients in the different BMI groups with natural cycle and an age of not more than 35 years old

Patients with a natural cycle and not more than 35 years old were divided into three groups according to their BMI: normal weight group (*n* = 440), overweight group (*n* = 178), and obese group (*n* = 197). The daily HCG hormone level and pregnancy conditions of patients in these three groups are presented in Table 1. The hormone levels of these three groups of patients were compared, and the results revealed that there was statistical significance for the hormone E_2_ level in these three groups of patients (*H* = 16.571, *P* < 0.001). Furthermore, it was found that the E_2_ level of patients in the obese group was lower than that of patients in the normal weight group, and the difference was statistically significant (*P* < 0.05), when compared between any two groups. This suggests that the difference was statistically significant (*H* = 17.903, *P* < 0.001) when comparing the hormone LH levels of these three groups of patients. Furthermore, the comparison between any two groups revealed that the LH level of patients in the obese group was lower than that of patients in the normal weight group. Therefore, the difference was statistically significant (*P* < 0.05). However, there was no statistical significance for the difference in hormone P in these three groups of patients by comparison (*H* = 1.534, *P* = 0.464). The pregnancy rate was 14.55% in the normal weight group, 10.67% in the overweight group, and 14.72% in the obese group, respectively. Similarly, there was no statistical significance for the difference in pregnancy rate among the three groups of patients by comparison (*X*^*2*^ = 1.812, *P* = 0.404). The specific details are presented in Table 1.

**Table 1:** Hormones and pregnancy in different body mass index groups in natural cycle pregnancy patients ≤35 years old

Grouping	Number of cases	HCG day E2	HCG day LH	HCG day P	Pregnancy condition	
					Not pregnant	Pregnant
Normal weight	440	1105.50 (808.83 ~ 1426.75)	34.34 (19.34 ~ 54.48)	2.42 (1.70 ~ 3.16)	376 (85.45%)	64 (14.55%)
Overweight	178	1017.00 (695.93 ~ 1347.08)	29.42 (16.96 ~ 50.87)	2.23 (1.72 ~ 3.03)	159 (89.33%)	19 (10.67%)
Obesity	197	927.30 (696.10 ~ 1201.00)^*^	25.32 (14.14 ~ 43.05)^*^	2.29 (1.67 ~ 2.91)	168 (85.28%)	29 (14.72%)
Statistic quantity		*H* = 16.571	*H* = 17.903	*H* = 1.534	*χ*^*2*^ = 1.812	
Parameters		*p* < 0.001	*p* < 0.001	*p* = 0.464	*p* = 0.404	

*P < 0.05

### Comparison of hormone and pregnancy for patients in the different BMI groups with an ovulation induction cycle and an age of not more than 35 years old

Patients with an ovulation induction cycle and an age of not more than 35 years were divided into three groups according to their BMI: normal weight group (*n* = 425), overweight group (*n* = 203), and obese group (*n* = 516). The hormone levels on HCG day and pregnancy conditions in the three groups are presented in Table 2. The hormone levels of these three groups of patients were compared, and the results revealed that the difference in hormone E_2_ level in these three groups of patients was statistically significant (*H* = 66.341, *P* < 0.001). Furthermore, it was found that the hormone E_2_ level was lowest in the obese group and highest in the normal weight group, when comparing between any two groups, and the difference in hormone E_2_ level for these different groups was statistically significant (*P* < 0.05). This suggests that the difference has statistical significance (*H* = 10.235, *P* = 0.006) when comparing the hormone LH levels of these three groups of patients. Furthermore, the comparison between any two groups revealed that the hormone LH level in the obese group was lower than that in the normal weight group, and the difference was statistically significant (*P* < 0.05). There was also statistical significance for the difference in hormone P of these three groups of patients by comparison (*H* = 1.534, *P* = 0.464). The pregnancy rate was 28.71% in the normal weight group, 29.56% in the overweight group, and 25.58% in the obese group, respectively. Similarly, there was no statistical significance for the difference in pregnancy rate among these three groups of patients by comparison (*X*^*2*^ = 1.694, *P* = 0.429). The specific details are presented in Table 2.

**Table 2:** Hormones and pregnancy in different body mass index groups in ovulation induction cycles ≤35 years old

Grouping	Number of cases	HCG day E2	HCG day LH	HCG day P	Pregnancy condition	
					Not pregnant	Pregnant
Normal weight	425	1538.00 (827.92 ~ 2828.50)	12.83 (8.14 ~ 23.26)	2.37 (1.69 ~ 3.18)	303 (71.29%)	122 (28.71%)
Overweight	203	1164.00 (694.30 ~ 2063.00)^*^	11.83 (7.58 ~ 24.38)	2.17 (1.61 ~ 2.91)	143 (70.44%)	60 (29.56%)
Obesity	516	933.45 (525.65 ~ 1607.75)^*#^	10.69 (6.96 ~ 19.01)^*^	2.11 (1.49 ~ 2.85)^*^	384 (74.42%)	132 (25.58%)
Statistic quantity		*H* = 66.341	*H* = 10.235	*H* = 12.912	*χ*^*2*^ = 1.694	
Parameters		*p* < 0.001	*p* = 0.006	*p* = 0.002	*p* = 0.429	

Note:*indicates *p* < 0.05 compared with normal weight, and # indicates *p* < 0.05 compared with overweight

### Comparison of hormone and pregnancy in patients in the different BMI groups with a natural cycle and an age of more than 35 years old

Patients with a natural cycle and an age of more than 35 years were divided into three groups according to their BMI: normal weight group (*n* = 90), overweight group (*n* = 83), and obese group (*n* = 81). The daily HCG hormone level and pregnancy conditions of these three groups of patients are presented in Table 3. The hormone levels of these three groups of patients were compared, and the results revealed that there was no statistical significance in the difference in hormone E_2_, LH and P levels of these three groups of patients (*P* > 0.05). The pregnancy rate was 12.22% in the normal weight group, 9.64% in the overweight group, and 11.11% in the obese group, respectively. Similarly, there was no statistical significance for the difference in pregnancy rate among these three groups of patients by comparison (*X*^*2*^ = 0.295, *P* = 0.863). The specific details are presented in Table 3.

**Table 3:** Hormones and pregnancy in different body mass index groups in natural cycle pregnancy patients > 35 years old

Grouping	Number of cases	HCG day E2	HCG day LH	HCG day P	Pregnancy condition	
					Not pregnant	Pregnant
Normal weight	90	1228.00 (795.30 ~ 1598.25)	23.53 (13.87 ~ 38.99)	1.80 (1.26 ~ 2.89)	79 (87.78%)	11 (12.22%)
Overweight	83	1072.00 (753.40 ~ 1468.00)	24.58 (12.96 ~ 45.54)	1.82 (1.23 ~ 2.75)	75 (90.36%)	8 (9.64%)
Obesity	81	1084.00 (713.80 ~ 1359.50)	22.35 (12.87 ~ 36.08)	1.73 (1.18 ~ 2.58)	72 (88.89%)	9 (11.11%)
Statistic quantity		*H* = 2.987	*H* = 1.040	*H* = 0.236	*χ*^*2*^ = 0.295	
Parameters		*p* = 0.225	*p* = 0.594	*p* = 0.889	*p* = 0.863	

### Comparison of hormone and pregnancy for patients in the different BMI groups with an ovulation induction cycle and an age of more than 35 years old

Patients with an ovulation induction cycle and an age of more than 35 years were divided into three groups according to their BMI: normal weight group (*n* = 26), overweight group (*n* = 26), and obese group (*n* = 54). The daily HCG hormone level and pregnancy conditions of these three groups of patients are presented in Table 4. The hormone levels of these three groups of patients were compared, and the results revealed that there was no statistical significance for the difference in hormone E_2_, LH and P levels of these three groups of patients (*P* > 0.05). The pregnancy rate was 15.38% in the normal weight group, 11.54% in the overweight group, and 7.41% in the obese group, respectively. Similarly, there was no statistical significance for the difference in pregnancy rate among these three groups of patients by comparison (*X*^*2*^ = 1.251, *P* = 0.535). The specific details are presented in Table 4.

**Table 4:** Hormones and pregnancy in patients older than 35 years old with different body mass index (BMI)

Grouping	Number of cases	HCG day E2	HCG day LH	HCG day P	Pregnancy condition	
					Not pregnant	Pregnant
Normal weight	26	1120.50 (677.65 ~ 2180.50)	15.98 (7.90 ~ 22.19)	1.65 (0.96 ~ 2.45)	22 (84.62%)	4 (15.38%)
Overweight	26	1063.35 (649.75 ~ 2693.75)	13.15 (8.30 ~ 26.46)	2.37 (1.55 ~ 3.10)	23 (88.46%)	3 (11.54%)
Obesity	54	1123.50 (653.50 ~ 1634.50)	15.62 (7.92 ~ 27.05)	1.90 (1.49 ~ 2.41)	50 (92.59%)	4 (7.41%)
Statistic quantity		*H* = 0.236	*H* = 0.105	*H* = 5.160	*χ*^*2*^ = 1.251	
Parameters		*p* = 0.889	*p* = 0.949	*p* = 0.076	*p* = 0.535	

### Analysis of the correlations between BMI and the different hormones

**Fig. 1:**
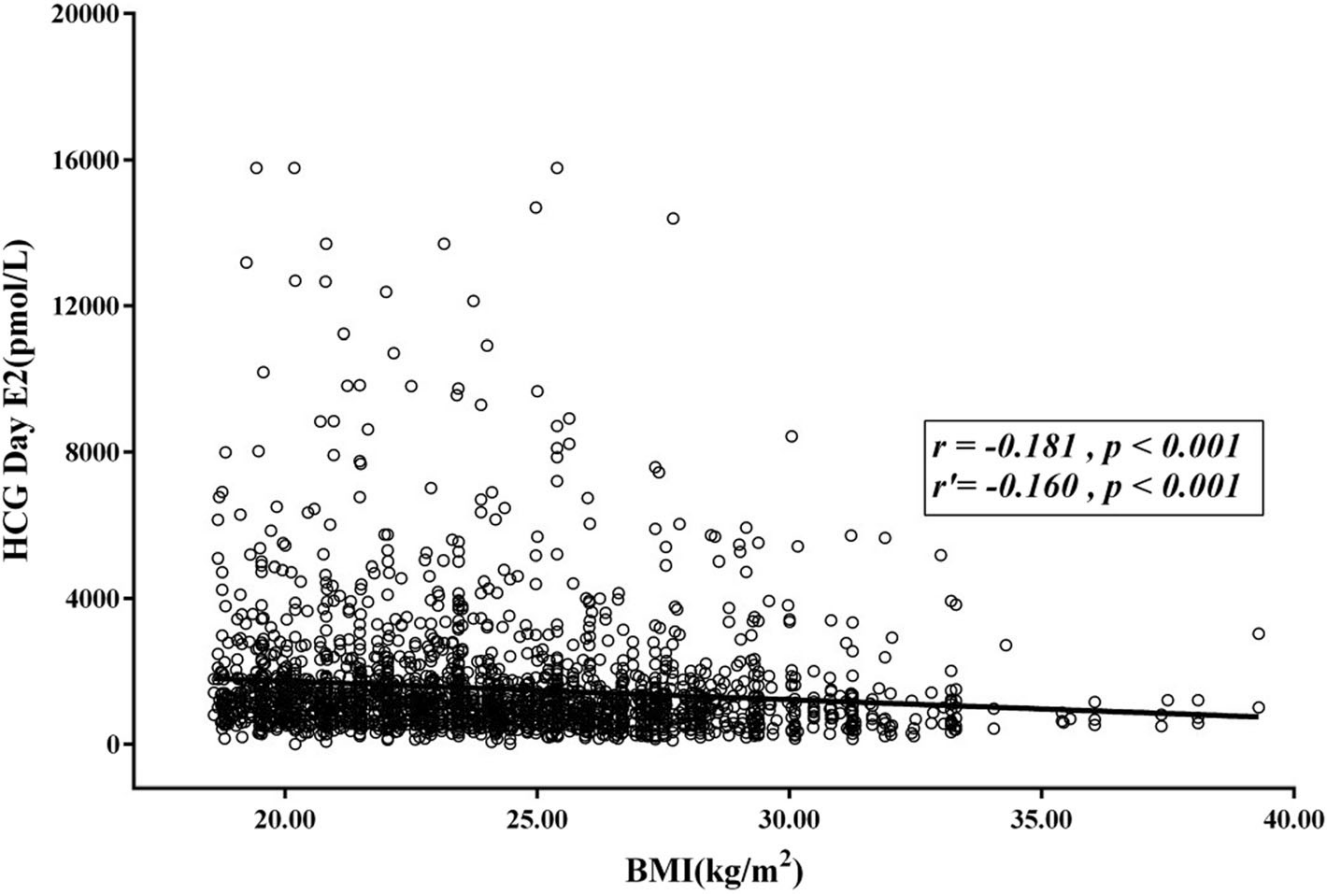
The scatter plot of the correlation between the BMI index and hormone E_2_. The correlation analysis between BMI and E_2_ on HCG day revealed that there was a negative correlation between BMI and E_2_ on HCG day. The higher the BMI index, the lower the level of E_2_ became. The correlation coefficient was − 0.181, the partial correlation number was − 0.160, and the *P*-value was < 0.001. The difference was statistically significant

**Fig. 2:**
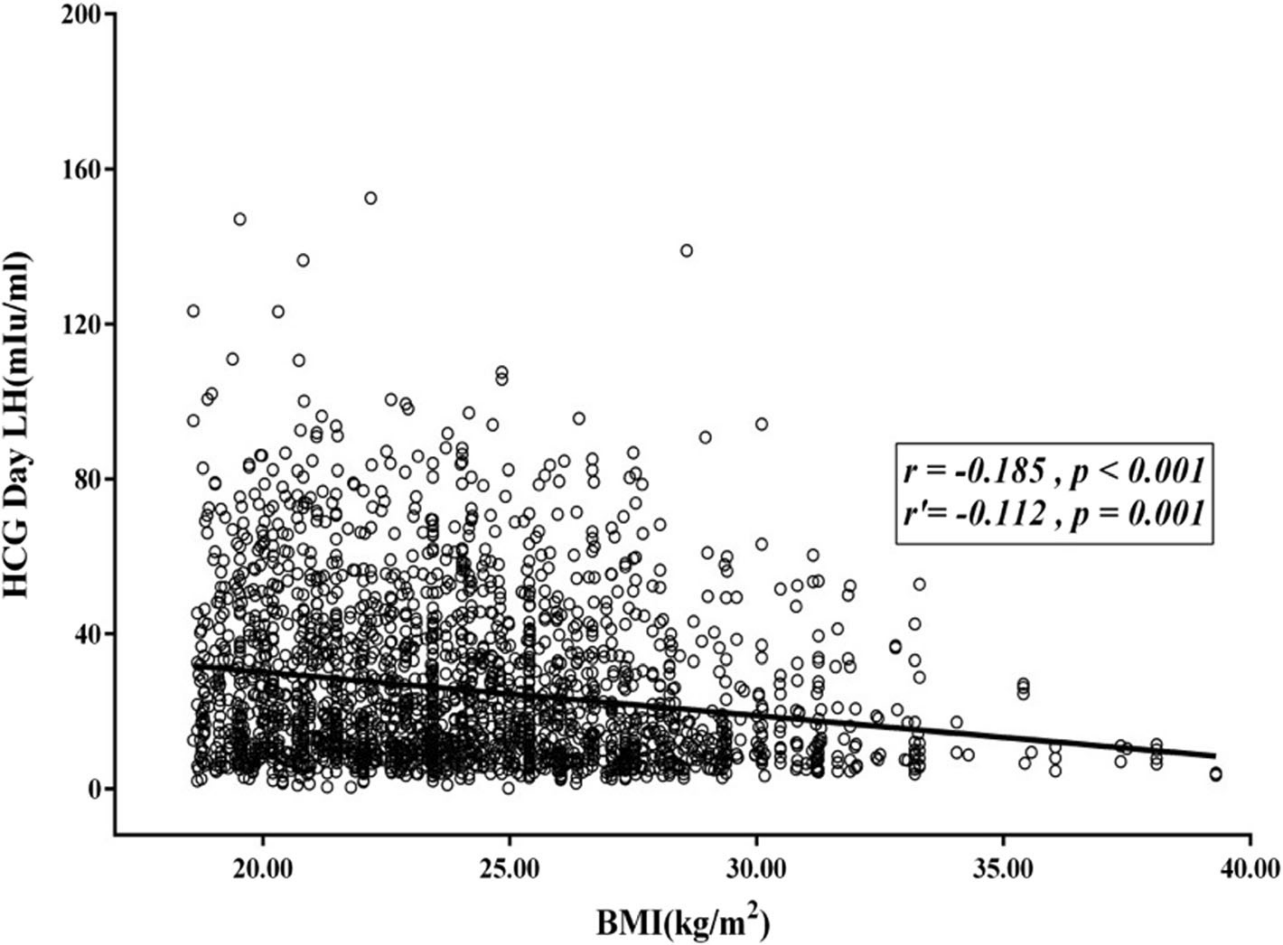
The scatter plot of the correlation between the BMI index and hormone LH. The correlation between BMI and LH on HCG day was analyzed. The results revealed that there was a negative correlation between BMI and LH. The higher the BMI index was, the lower the LH level became. The correlation coefficient was − 0.185, the partial correlation number was − 0.112, and the *P*-values were ≤ 0.001. The difference was statistically significant

The correlation between BMI and each hormone was analyzed for all patients, and the results are present in Table 5. The findings revealed that BMI is negatively correlated with the levels of hormone E_2_, LH and P on HCG day. That is, the higher the BMI was, the lower the levels of hormone E_2_, LH and P in HCG day were. After mastering the age and assisted reproduction conditions, the correlation analysis revealed that BMI was negatively correlated with the levels of hormone E_2_ and LH, with partial correlation coefficients of − 0.160 and − 0.112, respectively. All these had statistical significance (*P* < 0.05). The scatter diagrams of the correlation between BMI and hormone E_2_ and LH are presented in Figs. 1 and 2, respectively.

**Table 5:** Correlation analysis between BMI index and different hormone

Correlation	HCG day E2	HCG day LH	HCG day P
BMI index			
Correlation coefficient *r*	−0.181	−0.185	−0.104
*P value*	< 0.001	< 0.001	< 0.001
Partial correlation coefficient *r’*	− 0.160	− 0.112	− 0.034
*P value*	< 0.001	0.001	0.093

## Discussion

The influence of female BMI on hormone level after IUI on HCG day is presented, as follows. For patients with a natural cycle and an age of less than 35 years old, the E_2_ concentration and LH level in serum in the normal weight group were higher than those in the obesity group (*P* < 0.05), but the P concentration in serum was not significantly affected (*P* > 0.05). For patients with an induced ovulation cycle and an age of less than 35 years old, the levels of E_2_, LH and P in serum in the normal weight group were higher than those in the overweight group and obesity group (*P* < 0.05), respectively. The correlation analysis between infertility and BMI revealed that the risk of infertility increases with the increase in BMI. In particular, as the BMI becomes higher than 23.9 kg/m^2^, the risk of infertility significantly increases [22].

In the present study, the BMI was negatively correlated with E_2_, LH and P on HCG day. That is, the higher the BMI was, the lower the levels of E_2_, LH and P on HCG day became. However, the BMI value had no significant influence on pregnancy outcome, which may be due to the small sample capacity. Therefore, it is necessary to expand the sample capacity in further studies.

In a normal menstrual cycle, E_2_ secretion follows a certain rule, and fits with the growth and development of follicles, as well as the ovulation process. After fertilization, oocyte and progesterone work together to provide a basis for embryo implantation [23]. The pregnancy outcome of IUI is affected by many factors, and E_2_ is an important monitoring index for COS. It can be observed in previous studies that compared the pregnancy outcomes with different fluctuation degrees of E_2_ before the HCG trigger was compared, when E_2_ declines before the HCG trigger, there would be no adverse effects on the clinical pregnancy rate, live births rate and abortion rate in the early stage. This shows that the pregnancy outcome would not be affected by the dynamic change in E_2_ before the HCG trigger. Based on previous studies, the present study further investigates the influence of BMI on the change in E_2_ on HCG day on the pregnancy outcome of IUI. These results show that when age is no more than 35 years old, regardless of whether the patient has a natural cycle or promoting cycle, the BMI of women has an effect on the changes in E_2_ on HCG day. The E_2_ level in the obese group was lower than that in the normal weight group. When the age was more than 35 years old, the BMI had no effect on the changes in E_2_ on HCG day, regardless of whether the patient has a natural cycle or ovulation induction cycle. This could be due to the altered hormone levels in women of over 35 years old, especially the reduced E_2_. Furthermore, BMI was negatively correlated with the hormone level on HCG day. That is, the higher the BMI was, the lower the level of E_2_ became. In the study conducted by Mittal et al., [24] it was found that a change in E_2_ may affect the quality of oocytes and embryos, and that a higher estrogen/follicle ratio can lead to high quality oocytes, suggesting that better pregnancy outcomes can be obtained with the increase in E_2_. The present study compared the effects of the rise or fall of E_2_ on HCG day on the pregnancy outcomes of women with different BMIs, and the results revealed that there was no significant difference in pregnancy outcomes of women with different ages and the BMIs after IUI, regardless of whether they have a natural cycle or ovulation induction cycle (*P* > 0.05). This suggests that the rise and fall of E_2_ on HCG day has no significant adverse effect on pregnancy outcome.

Wu et al. [25] considered that excessively high E_2_ levels do not affect the final outcome of assisted pregnancy. However, Mitwally et al. [26] considered that a higher E_2_ would affect endometrial receptivity, and oocyte and embryo quality. Furthermore, if patients are more than 35 years old, their pregnancy rate for artificial-assisted pregnancy would more easily be affected by higher E_2_. The E_2_ level in the present study did not affect the pregnancy outcome, and there was no significant correlation between these two.

LH is a gonadotropin, which is needed by follicle development towards the dominant follicle [27], and the estrogen secretion simulated by LH is conducive to the maturation of the oocyte cytoplasm and egg membrane (refers to the zona pellucida and corona radiata). The main function of the LH pulse peak is to start the meiosis. Primary oocytes begin the meiosis and discharge the first polar body, thereby forming secondary oocytes and stopping at the meiosis metaphase, which in turn promotes the luteinization of the follicular wall, and finally stimulates ovulation. LHR is expressed by granulosa cells in the middle and late stage of the follicular stage. LH acts on the contact relaxation between granulosa cells and oocytes caused by follicles, in order to promote oocyte maturation and estrogen synthesis.

## Conclusion

In women less than 35 years old, the BMI affects the E_2_, LH and *P* values on the trigger day of artificial insemination. Furthermore, the higher the BMI was, the lower the levels of E_2_, LH and P became. However, a lower BMI does not obviously improve the clinical pregnancy rate of female patients treated by IUI. Hence, there is a need to conduct larger randomized controlled trials, in order to determine whether female BMI can change the pregnancy outcome.

## Data Availability

Not applicable.

## Abbreviations

BMI: Body mass index
IUI: Intrauterine insemination
HCG: Human Chorionic Gonadotropin
WHO: World Health Organization
HPO: Hypothalamic-pituitary-ovarian axis
SD: Standard deviation
